# Nonalcoholic fatty liver disease and hepatic fibrosis among perinatally HIV-monoinfected Asian adolescents receiving antiretroviral therapy

**DOI:** 10.1371/journal.pone.0226375

**Published:** 2019-12-19

**Authors:** Tavitiya Sudjaritruk, Torsak Bunupuradah, Linda Aurpibul, Pope Kosalaraksa, Nia Kurniati, Jiratchaya Sophonphan, Panruethai Trinavarat, Pannee Visrutaratna, Jiraporn Srinakarin, Nataruks Chaijitraruch, Thanyawee Puthanakit, on behalf of the NAFLD Study Group

**Affiliations:** 1 Department of Pediatrics, Faculty of Medicine, Chiang Mai University, Chiang Mai, Thailand; 2 Research Institute for Health Sciences, Chiang Mai University, Chiang Mai, Thailand; 3 HIV-NAT, The Thai Red Cross AIDS Research Centre, Bangkok, Thailand; 4 Department of Pediatrics, Faculty of Medicine, Khon Kaen University, Khon Kaen, Thailand; 5 Department of Child Health, Cipto Mangunkusumo General Hospital, Jakarta, Indonesia; 6 Department of Radiology, Faculty of Medicine, Chulalongkorn University, Bangkok, Thailand; 7 Department of Radiology, Faculty of Medicine, Chiang Mai University, Chiang Mai, Thailand; 8 Department of Radiology, Faculty of Medicine, Khon Kaen University, Khon Kaen, Thailand; 9 Department of Pediatrics, Faculty of Medicine, Chulalongkorn University, Bangkok, Thailand; 10 Center of Excellence in Pediatric Infectious Diseases and Vaccine, Chulalongkorn University, Bangkok, Thailand; Institute of Hepatology, Foundation for Liver Research, UNITED KINGDOM

## Abstract

To assess and compare the prevalence of persistent hepatic abnormalities, including nonalcoholic fatty liver disease (NAFLD) and/or hepatic fibrosis, among perinatally HIV-monoinfected Asian adolescents with history of abnormal hepatic enzymes to those without, using noninvasive diagnostic tools. A multicenter cohort study was conducted in Thailand and Indonesia. Adolescents aged 10–25 years who were on antiretroviral treatment (ART), had virologic suppression (HIV RNA<400 copies/mL within the past 6 months), and had no history of chronic hepatitis B/C infection were enrolled. Participants were pre-classified into 2 subgroups (1:1 ratio) as participants with history of elevated *versus* normal aminotransferase enzymes. NAFLD was defined as hepatic steatosis (any severity) evaluated by liver ultrasonography. Significant hepatic fibrosis was defined as liver stiffness ≥7.4 kPa evaluated by transient elastography. Participants who met the criteria for protocol-defined NAFLD and/or hepatic fibrosis were re-assessed to evaluate disease progression (persistent *versus* transient hepatic abnormalities) at one year later. Of 120 participants, 62 (51.7%) were male, 7 (5.8%) had central obesity, and 19 (15.8%) had insulin resistance (homeostasis model assessment of insulin resistance [HOMA-IR] >3.16). At enrollment, the median age and duration of ART (IQR) were 17.0 (14.6–19.2) years and 10.5 (7.1–12.0) years, respectively. Persistent hepatic abnormalities were identified in 5/60 participants listed in the group having history of elevated aminotransferases, corresponding to the prevalence of 8.3% (95% CI: 2.8–18.4%), whereas none (0/60) were among the group having history of normal hepatic enzymes. All 5 participants had persistent aminotransferase elevation (≥2 episodes within the past 12 months). Baseline alanine aminotransferase (ALT) >30 U/L (adjusted odds ratio [aOR]: 29.1; 95% CI: 1.7–511.8), and HOMA-IR >3.16 (aOR: 17.9; 95% CI: 1.1–289.7) were independently associated with persistent hepatic abnormalities. Among perinatally HIV-monoinfected Asian adolescents with history of elevated aminotransferase enzymes, persistent hepatic abnormalities are not uncommon. Screening for liver complications by noninvasive diagnostic tools might be considered in at risk individuals, including those with persistent ALT elevation and insulin resistance.

## Introduction

In the combination of antiretroviral therapy (cART) era, mortality associated with HIV and opportunistic infections have dramatically declined, leading to the significantly improved survival for HIV-infected children and adults [1,2]. However, long-term, non-AIDS-related complications have become increasingly recognized in these survivors. Liver disease has emerged as one of the leading causes of death among HIV-infected persons [2]. The increased burden of liver-related mortality, in both adults and children living with HIV, has drawn a great deal of clinical attention.

Over the last decade, the number of obese adolescents has risen substantially. In 2016, the prevalence of obesity among general adolescents was approximately 6% worldwide, and 2% in Southeast Asia [3]. As a consequence, nonalcoholic fatty liver disease (NAFLD), an important complication of obesity, has been increasingly recognized in this population with a prevalence of 11–17% [4]. NAFLD is a clinical-pathological syndrome characterized by the presence of excessive fat in hepatocytes in individuals without significant alcohol consumption [5]. NAFLD has been identified in 31–65% of HIV-monoinfected adults, and was independently associated with male sex, obesity, dyslipidemia, insulin resistance, and elevated serum alanine aminotransferase (ALT) to aspartate aminotransferase (AST) ratio [6–9]. Hepatic fibrosis is a consequence of NAFLD which may progress to cirrhosis and eventually hepatocellular carcinoma (HCC) [10]. This condition has been found in 15–42% of HIV-monoinfected individuals, and was linked to diabetes, and increased ALT and gamma-glutamyl transferase (GGT) [8,11]. However, the impact of antiretroviral agents, particularly nucleoside reverse transcriptase inhibitors (NRTIs) and protease inhibitors (PIs) on NAFLD [6–8] and hepatic fibrosis [8,11] among HIV-monoinfected adults are still controversial.

The gold standard for diagnosis of NAFLD and hepatic fibrosis is liver biopsy [5]. However, the invasiveness, potential risks, and impracticality of this procedure have prompted the exploration of alternative noninvasive diagnostic approaches, including radiological techniques (e.g., liver ultrasonography, controlled attenuation parameter [CAP], transient elastography [TE], computerized tomography [CT], magnetic resonance imaging [MRI], magnetic resonance elastography [MRE]), and liver biomarkers (e.g., cytokeratin-18 fragments [CK-18], AST-to-platelet ratio index [APRI], fibrosis-4 [FIB-4]) to evaluate hepatic steatosis, fibrosis, and cirrhosis [12–16]. Liver ultrasonography is a commonly used tool for NAFLD assessment because of its wide availability and absence of radiation exposure, but it is not good at detecting hepatic fibrosis. CAP is another measurement for NAFLD which is able to quantify the degree of steatosis and is operator-independent. In a previous study, CAP greater than or equal 249 dB/m demonstrated a good diagnostic accuracy for hepatic steatosis detection in children and adolescents with a sensitivity of 72%, a specificity of 98–100%, and an area under the receiver operating characteristic (AUROC) curve of 0.84 [17]. CT is seldom used for evaluating NAFLD, particularly in children, because of high radiation exposure. TE is a useful technique for detecting hepatic fibrosis since it demonstrates a good correlation with liver histology and liver biomarkers. In a recent meta-analysis, TE showed a highly accurate diagnostic performance for the diagnosis of significant hepatic fibrosis in children and adolescents with a sensitivity, a specificity, and an AUROC curve of 95%, 90%, and 0.96, respectively [18]. MRI/MRE is an advanced technique for the assessment of NAFLD and hepatic fibrosis with the greatest accuracy, but is not widely used because of its high cost [19]. CK-18 is a liver biomarker which is used to distinguish nonalcoholic steatohepatitis (NASH) from hepatic steatosis. A previous study demonstrated that an AUROC curve of CK-18 M30 for predicting NASH was 0.85–0.93 in children and adolescents [20,21]. These noninvasive measurements have been extensively investigated in HIV-infected adults [6–8,11,22,23], with limited studies in HIV-infected youth [24].

Elevation of aminotransferase enzymes are commonly observed in HIV-infected populations receiving cART, even in the absence of viral hepatitis coinfection [25,26]. Nevertheless, the association of aminotransferase elevation and significant liver diseases has not been well documented. Previous studies found that HIV-monoinfected adults with raised aminotransferase levels had a high prevalence of NASH (26–53%) and hepatic fibrosis (63%) [9,27], but there are still lack of data among adolescents.

The quiescent evolution of NAFLD and hepatic fibrosis towards irreversible harmful outcomes, specifically cirrhosis and end-stage liver diseases, highlights the importance of these complications in the management of people living with HIV infection and lifelong treatment. Since hepatic enzymes are inexpensive, widely available, and easily accessible biomarkers which might reflect underlying hepatic comorbidities, this study aimed to assess and compare the prevalence of persistent hepatic abnormalities, including NAFLD and/or hepatic fibrosis, among perinatally HIV-monoinfected adolescents with history of abnormal hepatic enzymes to those without, using noninvasive diagnostic tools.

## Methods and measurements

### Study design and participants

We conducted a multicenter cohort study in four clinical research sites: three in Thailand and one in Indonesia. Perinatally HIV-infected adolescents aged 10–25 years who were on ART, had a documentation of virologic suppression (HIV RNA <400 copies/mL within the past 6 months), and had no documented history of chronic hepatitis B or C infection were enrolled. Since we aimed to compare the prevalence of persistent hepatic abnormalities among adolescents who had abnormal hepatic enzymes to those without, eligible participants were pre-classified into 2 subgroups (ratio of 1:1), according to their past results of serum hepatic transaminases as Subgroup 1: participants with history of elevated aminotransferase levels if they had AST >50 U/L and/or ALT >30 U/L at least once within the past 12 months; and Subgroup 2: participants with history of normal aminotransferase levels. Briefly, we consecutively identified participants with history of elevated aminotransferases at each site. Then, participants with history of normal aminotransferase levels who had similar age (+/- 12 months) and sex across all sites were selected. The exclusion criteria were self-report significant alcohol consumption (>21 or >14 drinks on an average per week over a 2-year period prior to enrollment for male and female, respectively), a history of autoimmune hepatitis or inherited liver disease, or receiving any drug or chemical agent demonstrating liver injury. The study was approved by the local Institutional Review Boards at all participating sites, including the Research Ethics Committee, Faculty of Medicine, Chiang Mai University (Approval number: 097/2014), the Human Experimentation Committee, Research Institute for Health Sciences, Chiang Mai University (Approval number: 7/2014), the Institutional Review Board, Faculty of Medicine, Chulalongkorn Univeristy (Approval number: 118/2014), the Khon Kaen University Ethics Committee for Human Research (Approval number: HE571099), and the Health Research Ethics Committee, Faculty of Medicine, Universitas Indonesia, Cipto Mangunkusumo Hospital (Approval number: 369/H2.F1/ETIK/2014). All participants and their caregivers provided written informed consent and assent, as appropriate, prior to study enrollment.

### Clinical assessments and data collection

Demographic and HIV-related characteristics were abstracted from medical records. Anthropometric assessments, including weight, height and waist circumference (WC), and complete physical examination were performed at study entry. For participants aged <15 years, WC was converted into age- and sex-standardized z-score, using Thai normative reference [28]. Central obesity was defined as WC z-score >2, or WC ≥90 and ≥80 cm for male and female aged ≥15 years [29]. BMI for participants aged <18 years was transformed to BMI percentile based on the US Centers for Disease Control and Prevention reference [30]. Overweight were defined as BMI >85^th^ percentile [31], or >25 kg/m^2^ for participants aged ≥18 years [32].

### Laboratory measurements

Liver profiles, including AST, ALT, and GGT; lipid profiles, including total cholesterol, high-density lipoprotein cholesterol, low-density lipoprotein cholesterol, and triglyceride; diabetic profiles, including fasting plasma glucose (FPG) and insulin; and hematologic profiles were performed at enrollment after an overnight fast. The normal levels of AST, ALT, and GGT were ≤50 U/L, ≤30 U/L, and ≤50 U/L, respectively. The homeostasis model assessment of insulin resistance (HOMA-IR; FPG [mg/dL] × fasting plasma insulin [μU/mL] / 405) was calculated, and the cutoff for insulin resistance was 3.16 [33].

### Noninvasive diagnostic measurements for NAFLD and hepatic fibrosis

#### Radiological techniques

Liver ultrasonography was performed by pediatric radiologists at each site who were blinded to participants’ clinical and laboratory data. The presence of hepatic steatosis was described as a diffuse increase in echogenicity of liver parenchyma. The severity was classified as mild, moderate and severe steatosis as shown in Table 1. To reduce inter-observer variability, the recorded ultrasound images of each participant were cross-read by all pediatric radiologists at the other participating sites. NAFLD was definitely diagnosed when site’s pediatric radiologist and at least one of the other site’s radiologists concordantly confirmed the presence of steatosis. The severity of hepatic steatosis, if discordant, was relied on the site’s radiologist.

**Table 1:** Severity of hepatic steatosis evaluated by noninvasive radiologic measurements.

Severity of hepatic steatosis	Noninvasive radiologic measurements	
	Liver ultrasonography [34]	Controlled attenuation parameter^a^ [35]
Mild	A slight and diffuse increase in hepatic parenchymal echoes with normal visualization of portal vein borders and diaphragm	>238 dB/m
Moderate	A moderate and diffuse increase in hepatic parenchymal echoes with slightly impaired visualization of portal vein borders and diaphragm	>259 dB/m
Severe	A severe and diffuse increase in hepatic parenchymal echoes with poor or no visualization of portal vein border, diaphragm, and posterior portion of the right lobe	>292 dB/m

^a^ The measurement was performed at the one-year follow-up in only participants with persistent hepatic abnormalities.

Transient elastography (TE; FibroScan^®^, Echosens, Paris, France) was performed on a fasting participant by a well-trained operator, following the manufacturer’s instructions. In brief, 10 measurements with the standard M probe were performed on each participant. TE results were considered reliable if the success rate was ≥60% and the interquartile range (IQR) was <30% of the median. The median value of successful measurements was reported as a hepatic stiffness level (kPa). For this study, we defined hepatic fibrosis as a TE ≥7.4 kPa because this cutoff has a high diagnostic accuracy (sensitivity 100% and specificity 92%) for the diagnosis of significant hepatic fibrosis (≥ F2 disease) in adolescent population [14].

#### Liver biomarkers

AST-to-platelet ratio index (APRI: AST [U/L] / upper limit of normal × 100 / platelet count [10^9^ L]) and fibrosis-4 (FIB-4: age [years] × AST [U/L] / platelet count [10^9^ L] × ALT^1/2^ [U/L]) scores were calculated. APRI scores >0.5 and FIB-4 scores >1.45 are suggestive of hepatic fibrosis [36,37]. Additionally, cytokeratin 18-apoptosin M30 fragments (CK-18 M30) were performed in a subgroup of participants (all participants with history of elevated aminotransferase enzymes, and participants who met the criteria for protocol-defined NAFLD and/or hepatic fibrosis), using enzyme-linked immunosorbent assay (Vivalavida AB, Nacka, Sweden) on Thermo Scientific^TM^ Varioskan^TM^ Flash (Thermo Fisher Scientific Oy, Vantaa, Finland).

### Definitions of NAFLD, hepatic fibrosis and persistent hepatic abnormalities

In this study, NAFLD was defined as evidence of hepatic steatosis (any severity), evaluated by liver ultrasonography. Hepatic fibrosis was defined as significant liver stiffness (TE ≥7.4 kPa), evaluated by TE. Participants who met the criteria for protocol-defined NAFLD and/or hepatic fibrosis were re-assessed for anthropometric parameters, physical statuses, laboratory measurements, and noninvasive liver evaluations at 1 year after their first assessments. Participants were defined as having persistent hepatic abnormalities if they still met the criteria indicating NAFLD and/or hepatic fibrosis by the one-year measurements, or were defined as having transient hepatic abnormalities if they had return-to-normal evaluations by the repeated measurements. Additionally, participants with persistent hepatic abnormalities were further investigated by liver MRI/MRE and CAP (CAP^TM^, Echosens, Paris, France) at the one-year follow-up. Briefly, CAP was performed simultaneously with TE, and the ultrasound attenuation (dB/m) represents percentage of steatosis. The severity of hepatic steatosis was described in Table 1.

### Statistical analysis

The prevalence of protocol-defined hepatic abnormalities (NAFLD and/or hepatic fibrosis) among participants with and without history of elevated aminotransferases were calculated. The prevalence, baseline clinical characteristics, laboratory parameters, and noninvasive liver evaluations of participants in each group were compared using chi-square and Wilcoxon rank sum tests for categorical and continuous variables, respectively. Similarly, the comparisons between participants who met the criteria of persistent *versus* transient hepatic abnormalities were performed. Additionally, laboratory parameters and liver biomarkers between initial and one-year follow-up evaluations were compared using McNemar’s test and Wilcoxon sign rank test for categorical and continuous variables, respectively.

Univariable logistic regression analyses were conducted to identify factors associated with the protocol-defined persistent hepatic abnormalities. Covariates which demonstrated a significance level of <0.05 were included in a multivariable model. The magnitudes of association were summarized with crude odds ratio (crude OR) and adjusted odds ratio (aOR), together with their 95% confidence intervals (CI), in the univariable and multivariable analyses, respectively. All statistical analyses were carried out using Stata statistical software, version 14. (StataCorp LP, College Station, TX). A two-sided *P* <0.05 was judged to be statistically significant.

## Results

### Participant characteristics and laboratory results

Between August 2014 and June 2015, 120 participants with the median age (IQR) of 17.0 (14.6–19.2) years were enrolled. Half of them (51.7%) were male, and 7 participants (5.8%) had central obesity. At enrollment, 38 participants (31.7%) were currently on PI-based regimens. Sixty-two participants (51.6%) had postnatal exposure to stavudine and 21 (17.5%) to didanosine. The median duration of ART and CD4 T-cell count (IQR) were 10.5 (7.1–12.0) years, and 725 (588–946) cells/mm^3^, respectively. Sixteen participants (13.5%) were current alcohol drinkers, with a median intake (IQR) of 0.8 (0.5–4.0) drinks/day. Other anthropometric parameters and HIV-related characteristics for participants with history of elevated and normal aminotransferase enzymes are summarized in Table 2. Additionally, among participants with history of elevated aminotransferase enzymes, 33 (55.0%) had persistent elevation (≥2 episodes within the past 12 months).

**Table 2:** Clinical characteristics and laboratory results of perinatally HIV-monoinfected adolescents, stratified by history of serum aminotransferase levels.

Characteristics^a^^,^^b^	Participants with history of elevated aminotransferase levels (n = 60)		Participants with history of normal aminotransferase levels (n = 60)	*P*	
***Anthropometric parameters***					
Body mass index (kg/m^2^)	19.5 (17.7–21.3)		18.7 (16.8–20.0)	0.07	
Overweight	7 (11.7)		3 (5.0)	0.07	
Waist circumference (cm) Central obesity	69.0 (63.0–72.0) 3 (5.0)		65.0 (61.0–71.0) 4 (6.7)	0.05 0.68	
***HIV-related characteristics***					
WHO clinical stage 3–4 prior to ART	32 (61.5)		25 (51.0)	0.29	
CD4 T-cell percentage prior to ART (%)	6.5 (2.0–20.0)		13.5 (5.0–20.1)	0.25	
Postnatal exposure to ART Stavudine Didanosine	32 (53.3) 13 (21.7)		30 (50.0) 8 (13.3)	0.79 0.25	
Current ART regimens NNRTI-based Boosted PI-based	40 (66.7) 20 (33.3)		42 (70.0) 18 (30.0)	0.59	
Duration of ART (years)	11.0 (7.2–11.9)		9.4 (7.0–12.0)	0.63	
Current CD4 T-cell count (cells/mm^3^)	694 (546–979)		771 (612–928)	0.33	
***Liver profiles***					
Frequency of AST/ALT measurements within 12 months prior to enrollment	3 (2–4)		2 (2–3)	0.004	
ALT (U/L) ALT >30 U/L	29 (21–39) 26 (43.3)		16 (12–21) 1 (1.7)	<0.001 <0.001	
AST (U/L) AST >50 U/L	24 (21–33) 2 (3.3)		21 (18–24) 0 (0)	<0.001 0.15	
Gamma GT (U/L) Gamma GT >50 U/L	47 (25–95) 27 (45.0)		33 (22–50) 15 (25.0)	0.03 0.02	
***Liver biomarkers***					
APRI	0.3 (0.2–0.3)		0.2 (0.1–0.2)	<0.001	
FIB-4	0.3 (0.2–0.4)		0.3 (0.2–0.4)	0.06	
***Metabolic profiles***					
Total cholesterol (mg/dL)	174 (161–196)		170 (154–197)	0.46	
HDL-cholesterol (mg/dL)	45 (39–67)		55 (44–67)	0.07	
LDL-cholesterol (mg/dL)	106 (86–125)		102 (88–116)	0.75	
Triglyceride (mg/dL)	87 (65–131)		86 (58–125)	0.51	
FPG (mg/dL)	83 (79–87)		81 (77–88)	0.43	
Insulin (mU/L)	7.7 (4.7–10.6)		8.2 (5.2–14.3)	0.23	
HOMA-IR HOMA-IR >3.16	1.5 (0.9–2.2) 8 (13.3)		1.5 (1.0–2.8) 11 (18.3)	0.32 0.48	

Abbreviations: ALT, alanine aminotransferase; APRI, aspartate aminotransferase-to-platelet ratio index; ART, antiretroviral treatment; AST, aspartate aminotransferase; FIB-4, fibrosis-4 score; FPG, fasting plasma glucose; Gamma GT, gamma-glutamyl transferase; HDL, high-density lipoprotein; HIV, human immunodeficiency virus; HOMA-IR, homeostasis model assessment of insulin resistance; LDL, low-density lipoprotein; NNRTI, non-nucleoside reverse transcriptase inhibitor; PI, protease inhibitor; WHO, World Health Organization.

^a^ Data presented as n (%) for categorical data and median (IQR) for continuous data. Chi-square test and Wilcoxon rank sum test were used to compare categorical and continuous data, respectively.

^b^ Data represented the characteristics at enrollment, unless otherwise specified.

For the laboratory parameters, participants in both groups had similar results, except for the liver profiles as they were the protocol-defined recruitment criteria for this study (Table 2). At enrollment, elevated ALT (ALT >30 U/L) was identified in 26 (43.3%) participants with history of elevated aminotransferase enzymes, and 1 (1.7%) participant with normal hepatic enzymes. Four participants (3.3%) had APRI >0.5; all were among the group with elevated aminotransferase levels, whereas none of the participants had FIB-4 >1.45. Insulin resistance (HOMA-IR >3.16) was observed in 8 (13.3%) and 11 (18.3%) participants among the groups having elevated *versus* normal aminotransferases, respectively. No participants were currently receiving antidiabetic or lipid-lowering medications in both groups.

### Prevalence of persistent hepatic abnormalities

**Fig 1:**
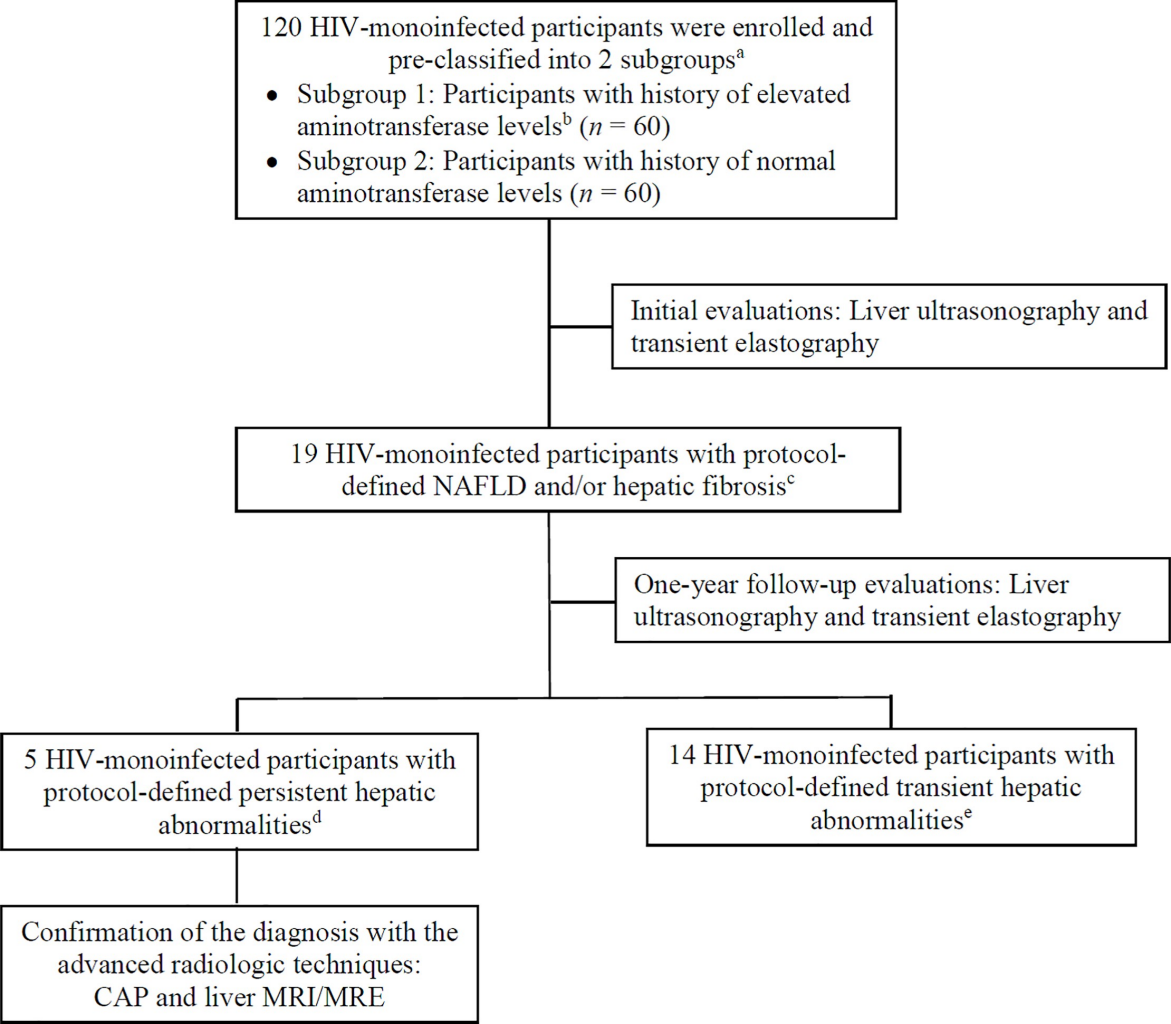
Flow chart of participants through the study. Abbreviations: CAP, controlled attenuation parameter; MRE, magnetic resonance elastography; MRI, magnetic resonance imaging; NAFLD, nonalcoholic fatty liver disease. ^a^ Participants in both groups were matched on age and sex in the ratio 1:1. ^b^ Participants were listed as having history of elevated aminotransferase levels if they had AST >50 U/L and/or ALT >30 U/L at least once within the past 12 months. ^c^ NAFLD was defined as an evidence of hepatic steatosis (any severity), evaluated by liver ultrasonography. Hepatic fibrosis was defined as significant liver stiffness (≥7.4 kPa), evaluated by transient elastography. ^d^ Persistent hepatic abnormalities was defined as an evidence of NAFLD and/or hepatic fibrosis at both initial and one-year follow-up evaluations. ^e^ Transient hepatic abnormalities was defined as an evidence of NAFLD and/or hepatic fibrosis at initial evaluation, but having a return-to-normal evaluation by the repeated measurements.

With the initial evaluations, 10 participants with history of elevated aminotransferases (16.7%; 5 with NAFLD, 4 with hepatic fibrosis, and 1 with both conditions) and 9 participants with history of normal hepatic enzymes (15.0%; 3 with NAFLD and 6 with hepatic fibrosis) demonstrated the evidences of hepatic abnormalities (*P* >0.05) (Fig 1). However, with the one-year follow-up measurements, only 5 participants in the group having history of elevated aminotransferase levels met the criteria for persistent hepatic abnormalities (3 with persistent NAFLD, 1 with persistent hepatic fibrosis, and 1 with both persistent NAFLD and hepatic fibrosis) (Fig 1), corresponding to the prevalence of 8.3% (95% CI: 2.8–18.4%). Additionally, all 5 participants had persistent aminotransferase elevation. None of the group of having normal hepatic enzymes demonstrated persistent hepatic abnormalities. Clinical information for each participant is shown in Table 3. Three participants (patient number 1, 2 and 3) had persistent moderate to severe hepatic steatosis by both liver ultrasonography and MRI. All of them had also insulin resistance (HOMA-IR >3.16), and 2 (patient number 1 and 3) had central obesity. Patient number 4 had persistent mild hepatic steatosis by ultrasonography; however, demonstrated normal evaluations by CAP and liver MRI. Patient number 5 had persistent hepatic fibrosis by TE, but did not undergo liver MRE due to patient incorporation. The other 14 participants demonstrated transient hepatic abnormalities, including 5 with history of elevated aminotransferases (2 with mild degree of NAFLD and 3 with hepatic fibrosis at the initial assessments), and 9 with history of normal hepatic enzymes (3 with mild degree of NAFLD and 6 with hepatic fibrosis at the initial evaluation) (Fig 1).

**Table 3:** Clinical information of perinatally HIV-monoinfected adolescents with persistent hepatic abnormalities.

**No.**	Age at enrollment (years)	Sex	BMI (kg/m^2^)	WC (cm)	Alcohol use (drink/day)	Current ART/ duration (years)	AST/ ALT (U/L)	HOMA-IR	Investigations at enrollment		Investigations at one-year follow-up			
									US: fatty liver grade	TE: stiffness (kPa)	US: fatty liver grade	TE: stiffness (kPa)	CAP (dB/m) / grade	Liver MRI/ MRE
1	23.4	M	36.2	114.0	4	TDF+3TC+EFV/ 11.4	87/ 160	4.8	Severe	14.0	Severe	12.3	332/ Severe	Moderate hepatic steatosis, and severe liver fibrosis
2	17.6	F	21.3	69.0	N	TDF+3TC+RPV/ 15.5	24/ 36	12.6	Severe	5.7	Severe	7.4	255/ Mild	Moderate hepatic steatosis, no liver fibrosis
3	21.6	M	31.8	102.0	N	TDF+FTC+EFV/ 11.7	39/ 67	3.6	Moderate	5.9	Severe	9.4	337/ Severe	Severe hepatic steatosis, no liver fibrosis
4	11.9	F	15.5	59.0	N	ZDV+3TC+NVP/ 1.6	31/ 36	2.8	Mild	5.7	Mild	3.7	201/ No	No evidences of hepatic steatosis and liver fibrosis
5	14.0	M	17.7	62.5	N	TDF+3TC+ATV/r/ 11.7	32/ 23	1.3	No	7.9	No	8.7	197/ No	-

Abbreviations: 3TC, lamivudine; ALT, alanine aminotransferase enzyme; ART, antiretroviral treatment; AST, aspartate aminotransferase enzyme; ATV/r, atazanavir/ritonavir; BMI, body mass index; CAP, controlled attenuation parameter; EFV, efavirenz; F, female; FTC, emtricitabine; HOMA-IR, homeostasis model assessment of insulin resistance; M, male; MRE, magnetic resonance elastography; MRI, magnetic resonance imaging; N, alcohol non-drinker; NVP, nevirapine; RPV, rilpivirine; TDF, tenofovir disoproxil fumarate; TE, transient elastography; US, ultrasonography; WC, waist circumference; ZDV, zidovudine.

### Comparisons of participants with persistent *versus* transient hepatic abnormalities

CK-18 M30 and insulin levels were significantly higher in participants with persistent hepatic abnormalities than those with transient abnormalities at enrollment (CK-18 M30: 203 *versus* 95 U/L; *P* = 0.01, insulin: 15.9 *versus* 8.7 mU/L; *P* = 0.04) and one-year follow-up (CK-18 M30: 336 *versus* 85 U/L; *P* = 0.03, insulin: 29.9 *versus* 10.3 mU/L; *P* = 0.02), while HOMA-IR was significantly greater at only one-year follow-up (6.0 *versus* 1.9; *P* = 0.02). Notably, there were no significant changes in all liver profiles and biomarkers from enrollment to one-year follow-up visit within each group of participants (*P* >0.05).

### Associated factors of persistent hepatic abnormalities

In the univariable model, central obesity (crude OR: 14.5; 95% CI: 2.0–107.5), baseline ALT >30 U/L (crude OR: 15.7; 95% CI: 1.7–146.8), and HOMA-IR >3.16 (crude OR: 9.1; 95% CI: 1.4–58.8) were associated with persistent hepatic abnormalities, whereas HIV-related characteristics, dyslipidemia, and liver biomarkers did not demonstrate any statistically significant associations (*P* >0.05) (Table 4). Both baseline ALT >30 U/L (aOR: 29.1; 95% CI: 1.7–511.8), and HOMA-IR >3.16 (aOR: 17.9; 95% CI: 1.1–289.7) remained independently associated with persistent abnormalities, while central obesity lost the significant association after adjusting for confounding factors (Table 4).

**Table 4:** Associated factors of persistent hepatic abnormalities among perinatally HIV-monoinfected Asian adolescents.

Parameters	Univariable analysis^a^		Multivariable analysis^a^	
	Cruded OR (95% CI)	*P*	Adjusted OR (95% CI)	*P*
Age (per 1 year increase)	1.23 (0.92–1.63)	0.17		
Male	0.61 (0.10–3.80)	0.60		
Overweight	8.92 (1.30–61.31)	0.03		
Central obesity	14.53 (1.96–107.52)	0.01	3.39 (0.17–69.96)	0.43
History of elevated aminotransferase levels	4.21 (0.46–38.86)	0.20		
WHO stage 3–4 prior to ART (*vs*. stage 1–2)	0.33 (0.03–3.32)	0.35		
CD4 T-cell <15% prior to ART	0.70 (0.09–5.16)	0.72		
Postnatal exposure to stavudine	0.22 (0.02–2.00)	0.18		
Postnatal exposure to didanosine	1.18 (0.12–11.08)	0.89		
Current PI used (*vs*. NNRTI used)	0.52 (0.06–4.31)	0.54		
Duration ART (per 1 year increase)	1.03 (0.79–1.34)	0.83		
Current CD4 T-cell ≥500 cells/mm^3^	1.14 (0.15–8.41)	0.90		
Baseline ALT >30 U/L	15.65 (1.67–146.83)	0.02	29.09 (1.65–511.76)	0.02
Baseline gamma GT >50 U/L	2.92 (0.47–18.23)	0.25		
APRI >0.5	9.17 (0.77–108.00)	0.11		
FIB-4	10.84 (0.07–1,773.00)	0.36		
HOMA-IR >3.16	9.09 (1.41–58.75)	0.02	17.93 (1.11–289.71)	0.04
Cholesterol ≥200 mg/dL	0.89 (0.10–8.32)	0.92		
HDL-cholesterol <40 mg/dL	2.23 (0.35–14.07)	0.39		
LDL-cholesterol ≥130 mg/dL	1.18 (0.12–11.08)	0.89		
Triglyceride ≥150 mg/dL	1.79 (0.19–17.14)	0.62		

Abbreviations: ALT, alanine aminotransferase; APRI, aspartate aminotransferase-to-platelet ratio index; ART, antiretroviral treatment; CI, confidence interval; CK-18 M30, cytokeratin 18-apoptosin M30 fragments; FIB-4, fibrosis-4; Gamma GT, gamma-glutamyl transferase; HDL, high-density lipoprotein; HOMA-IR, homeostasis model assessment of insulin resistance; LDL, low-density lipoprotein; NNRTI, non-nucleoside reverse transcriptase inhibitor; OR, odds ratio; PI, protease inhibitor; WHO, World Health Organization.

^a^ Univariable and multivariable logistic regression analyses were performed to identify factors associated with persistent hepatic abnormalities among perinatally HIV-monoinfected adolescents.

## Discussion

Using noninvasive diagnostic tools, including liver ultrasonography and TE, 8.3% of our perinatally HIV-monoinfected Asian adolescents with history of elevated aminotransferase levels met the criteria for protocol-defined persistent NAFLD and/or hepatic fibrosis. HIV-infected adolescents with normal aminotransferase enzymes are less likely to have persistent hepatic abnormalities. Therefore, in the clinical practice with high volume of patients, screening for these hepatic co-morbidities might be considered in adolescents with persistent ALT elevation plus metabolic risk factors, such as insulin resistance, to early detect the abnormalities and prevent the progression to irreversible harmful outcomes.

From the initial evaluation, the prevalence of NAFLD among our HIV-infected adolescents was 8% which was lower than that of HIV-uninfected adolescents (11–17%) [4,38]. Additionally, our prevalence was much lower than that demonstrated in a range of 31–55% among HIV-monoinfected US, Italian, Canadian, and Greek adults [6–8,39,40]. A recent systematic review and meta-analysis showed that a pooled prevalence of NAFLD, based on imaging studies, among HIV-monoinfected adults was 35% [41]. Focusing on our adolescents with history of elevated aminotransferases, 10% of them had NAFLD which was similarly much lower than a prevalence of 71% among HIV-monoinfected UK adults with persistent elevated aminotransferases [42]. This might be explained by the lower proportion of obesity and metabolic derangement in our adolescents, as well as the variation in definitions and the heterogeneity of diagnostic tools adopted for NAFLD.

Hepatic fibrosis is a critical consequence of NAFLD which is associated with liver-related mortality. In this study, 9% of adolescents had significant hepatic fibrosis (TE ≥7.4 kPa) in the first assessment which was lower than that observed in HIV-monoinfected adults [8,11,40–43]. In a recent systematic review and meta-analysis, a pooled prevalence of significant hepatic fibrosis, based on liver histology, was 22% among HIV-monoinfected US, Canadian, French, Italian, Japanese, and Chinese adults [41]. In addition, a recent UK study showed the 21% prevalence of significant hepatic fibrosis (TE ≥7.4 kPa) among their HIV-monoinfected adults with persistent raised aminotransferases [42], which was much greater than the 8% prevalence in our adolescents with history of elevated aminotranferases. Hepatic fibrosis usually takes years to develop; therefore, the prevalence is expected to be lower in adolescents compared with adults.

With the one-year follow-up, we noted that 8% of our adolescents with history of elevated aminotransferases demonstrated the evidence of persistent hepatic abnormalities. Meanwhile, none of the adolescents with history of normal hepatic enzymes showed such hepatic abnormalities. As participants with history of elevated hepatic enzymes had more frequent laboratory measurements compared with those having normal enzyme levels, selection bias might be occurred because participants classified in the former subgroup would be more likely to have hepatic abnormalities than those in the latter subgroup. Although, in this study, we did not perform liver biopsy, a benchmark for diagnosis of NAFLD and hepatic fibrosis, 3 participants (patient number 1, 2 and 3) were likely to have hepatic diseases since several evaluations, both conventional and advanced, concordantly showed the significant and persistent abnormalities. Moreover, such participants had insulin resistance and central obesity. For patient number 4, the observed persistent NAFLD might be due to measurement error of liver ultrasonography because the abnormalities did not demonstrate by CAP and liver MRI. For patient number 5, who demonstrated persistent hepatic fibrosis without evidences of NAFLD, we hypothesized that this might be attributed to measurement error of TE or other causes of hepatic fibrosis that were not investigated under this study. Liver MRI/MRE might provide more information for this patient, but were not conducted due to patient incorporation. For adolescents with transient hepatic abnormalities, we could not conclude whether this is the observational error of measurement or the actual disease regression. The advanced techniques such as liver MRI/MRE or liver histopathology would help confirming the diagnosis, but unfortunately were not performed in these individuals.

In this study, elevated ALT was independently associated with protocol-defined persistent hepatic abnormalities. Previous studies in HIV-monoinfected adults found that ALT elevation was correlated with NASH and significant hepatic fibrosis [8,9,27]. Furthermore, we noted that all adolescents with persistent hepatic abnormalities had at least two episodes of aminotransferase elevation within the past 12 months. So, although single ALT elevation might be attributed to measurement variation, persistent hepatic enzyme elevation might be used as a clue for further investigations that yield the diagnosis in clinical practices. However, there are some controversial evidence showing that elevated ALT might not be a good indicator of NAFLD in adolescents [38]. Therefore, larger clinical studies are needed to better establish the diagnostic accuracy of ALT for detecting NAFLD in HIV-infected adolescent population.

We also found that insulin resistance was associated with persistent hepatic abnormalities in our adolescents. Insulin resistance is the pathophysiological hallmark of NAFLD and hepatic fibrosis. It reduces insulin sensitivity at the levels of adipose tissue and liver, causing an accelerated hepatic fatty acid synthesis and an increased intrahepatic fat accumulation, which consequently results in hepatic steatosis, inflammation, and fibrosis [6,44]. The tight association between insulin resistance and hepatic abnormalities in HIV-infected populations was supported by several evidence. Two cross-sectional studies in HIV-monoinfected US adults with elevated aminotransferases demonstrated that insulin resistance was associated with NASH and hepatic fibrosis [9,22].

Postnatal exposure to NRTIs, particularly stavudine and didanosine, is a common factor associated with NAFLD and hepatic fibrosis among HIV-infected individuals [7,45]. The mechanism of the linkage is that NRTI directly induce mitochondrial toxicity [46]. In a previous study among HIV-monoinfected Italian adults, previous NRTI exposure was independently associated with NAFLD. Importantly, the risk of NAFLD in this population was increased 1.12 times for each year of NRTI exposure [7]. Additionally, treatment with didanosine was found to be a significantly associated factor of hepatic fibrosis among HIV-monoinfected Spanish adults [45]. Nevertheless, our study did not note the linkage between NRTI exposure and persistent hepatic abnormalities, including NAFLD and/or hepatic fibrosis. This might be due to a short duration of exposure to these drugs (1.9 years for stavudine and 3.4 years for didanosine) among our participants compared to the previous study [7].

Noninvasive liver biomarkers, such as APRI and FIB-4, have been well studied as the surrogate markers of hepatic fibrosis in HIV-infected children and adolescents [24]. However, this study did not demonstrate any statistically significant associations between these biomarkers and persistent hepatic abnormalities. This might be attributed to the low number of participants with abnormal APRI (3%) and FIB-4 (0%), as majority of them had no or mild degree hepatic fibrosis, resulting in the insufficient power to detect such associations. Additionally, we noted that participants with persistent hepatic abnormalities had greater CK-18 M30 levels compared to those with transient abnormalities at enrollment and one-year follow-up visits. This is similar to the previous studies which found that CK-18 M30 levels were significantly higher in youth with NAFLD/NASH than those without such hepatic abnormalities [20,21].

The adverse outcomes of NAFLD and hepatic fibrosis include the occurrences of cirrhosis, HCC and liver-related deaths. Several studies reported that NAFLD and hepatic fibrosis are significant risk factors for the development of cirrhosis and HCC [47]. Additionally, a previous study found that HIV-uninfected US adults with NAFLD had significantly increased overall mortality compared with general population of the same age and sex [48]. Promptly providing the appropriate managements at the early stage of NAFLD, including weight reduction, diet modification, physical exercise, and treatment with antidiabetic medications have demonstrated benefits in prevention or delay harmful complications in general population, but the evidences in HIV-infected individuals are still scarce [49].

To the best of our knowledge, this is the first study evaluating hepatic co-morbidities by noninvasive diagnostic measurements among HIV-infected adolescents in resource-constrained settings. Additionally, we use stringent criteria to define the persistent hepatic abnormalities, and confirm the diagnosis by several imaging modalities, including advanced techniques such as CAP and liver MRI/MRE. Nevertheless, this study has some limitations that should be acknowledged. Since NAFLD and hepatic fibrosis are long-term, progressive complications, evaluating such conditions while these individuals are adolescents might be too early to observe significant clinical outcomes. The prospective research with greater length of follow-up is required. In addition, using noninvasive radiologic measurements might contain some limitations, e.g., degraded image quality and reduced accuracy of liver ultrasonography and TE when perform in obese participants. Furthermore, since we performed the advanced radiologic techniques, including CAP and liver MRI/MRE, in only participants with persistent hepatic abnormalities at one-year follow-up, this might limit our ability to confirm hepatic abnormalities in the rest of the cohort. Also, a small number of participants, particularly those with persistent hepatic abnormalities, might limit our power to make a strong conclusion for this study. Finally, since this study was conducted in Asian adolescents whom might have distinct risk of metabolic disorders, our findings may have limited generalizability to populations in other regions.

## Conclusions

Among perinatally HIV-monoinfected Asian adolescents with history of elevated aminotransferase enzymes, persistent hepatic abnormalities, including NAFLD and/or hepatic fibrosis, are not uncommon. Persistent ALT elevation and insulin resistance appear to be associated with persistent hepatic abnormalities. Noninvasive diagnostic assessments might be considered in these at risk individuals to early detect significant hepatic complications. Future clinical studies with a larger sample size are needed to provide more evidence to make a recommendation on hepatic comorbidity screening for this population.

### The NAFLD study group

Faculty of Medicine and Research Institute for Health Sciences, Chiang Mai University, Chiang Mai, Thailand: Sudjaritruk T, Aurpibul L, Chotecharoentanan T, Wongnum N, Rungruengthanakit K, and Suwannamas N;

HIV-NAT, the Thai Red Cross AIDS Research Centre, Bangkok, Thailand: Puthanakit T, Bunupuradah T, Sophonphan J, Thamsala S, Pitimahajanaka T, Suwanlerk T, Thongpunchang B; Ubolyam S, Mahanontharit A, Laopraynak N, and Jaimulwong T;

Srinagarind Hospital, Khon Kaen University, Khon Kaen, Thailand: Kosalaraksa P, Sopharuk C, and Tharnprisan P;

Cipto Mangunkusumo General Hospital, Jakarta, Indonesia: Kurniati N, Wicaksana P,

## Supporting information

S1 Table NAFLD study dataset. (XLSX)
